# The contribution and interplay of implicit and explicit processes on physical activity behavior: empirical testing of the physical activity adoption and maintenance (PAAM) model

**DOI:** 10.1186/s12889-024-18589-5

**Published:** 2024-05-06

**Authors:** Darko Jekauc, Ceren Gürdere, Chris Englert, Tilo Strobach, Gioia Bottesi, Steven Bray, Denver Brown, Lena Fleig, Marta Ghisi, Jeffrey Graham, Mary Martinasek, Nauris Tamulevicius, Ines Pfeffer

**Affiliations:** 1 Institute of Sports and Sports Science, Karlsruhe Institute of Technology, Karlsruhe, Germany; 2 Department of Psychology, Bilkent University, Ankara, Turkey; 3 Institute of Sports Sciences, Goethe University Frankfurt, Frankfurt, Germany; 4 Medical School Hamburg, Institute of Cognitive and Affective Neuroscience (ICAN), Hamburg, Germany; 5 Department of General Psychology, University of Padova, Padova, Italy; 6 U.O.C. Hospital Psychology, University-Hospital of Padova, Padova, Italy; 7 Department of Kinesiology, McMaster University, Hamilton, Canada; 8 Department of Psychology, The University of Texas at San Antonio, San Antonio, USA; 9 Medical School Berlin, Department of Psychology, Berlin, Germany; 10 Department of Kinesiology, Recreation, and Sport Studies, The University of Tennessee, Knoxville, USA; 11 Department of Health Sciences and Human Performance, The University of Tampa, Tampa, USA

**Keywords:** Physical activity, Intention, Self-regulation, Executive functions, Habit, Affect

## Abstract

The adoption and maintenance of physical activity (PA) is an important health behavior. This paper presents the first comprehensive empirical test of the Physical Activity Adoption and Maintenance (PAAM) model, which proposes that a combination of explicit (e.g., intention) and implicit (e.g., habit,, affect) self-regulatory processes is involved in PA adoption and maintenance. Data were collected via online questionnaires in English, German, and Italian at two measurement points four weeks apart. The study included 422 participants (*M*_age_= 25.3, *SD*_age_= 10.1; 74.2% women) from Germany, Switzerland, Italy, Canada, and the U.S. The study results largely supported the assumptions of the PAAM model, indicating that intentions and habits significantly mediate the effects of past PA on future PA. In addition, the effect of past PA on future PA was shown to be significant through a mediation chain involving affect and habit. Although the hypothesis that trait self-regulation moderates the intention-behavior relationship was not supported, a significant moderating effect of affect on the same relationship was observed. The results suggest that interventions targeting both explicit and implicit processes may be effective in promoting PA adoption and maintenance.

## Introduction

Physical activity (PA) is an essential component of a healthy lifestyle and has been shown to have numerous health benefits [1]. Regular PA is associated with a reduced risk of developing chronic diseases such as cardiovascular disease [2], type 2 diabetes [3], and certain types of cancer [4]. In addition, PA is also beneficial for mental health and it can improve mood, reduce anxiety and depression [5, 6], and improve cognitive functions [7, 8]. Despite the well-documented benefits of PA, a large proportion of the population is not sufficiently active [9], and a significant proportion of individuals struggle to maintain regular PA behaviors [10, 11]. This lack of sufficient PA is a major public health concern because it contributes to the burden of chronic disease and related health care costs [12, 13]. Therefore, promoting the adoption and maintenance of PA is a key public health priority to improve health-related outcomes and reduce healthcare costs.

Because of the lack of sufficient PA, there is a need for theory-based prevention and health promotion programs to encourage PA adoption and maintenance. Most classical theoretical models used to explain PA, such as the theory of planned behavior [14] or the social cognitive theory [15], assume that intention (i.e., motivation for a specific behavior) is the most important predictor of behavior. Empirical studies support the importance of intentions in the implementation of PA [16]. Despite this, a notable discrepancy persists between individuals’ intentions and their actual PA behavior, with the absence of empirical evidence to validate the supremacy of rational processes, such as intention, over other potential determinants of behavior [17]. Consequently, meta-analytical findings have illuminated that a substantial proportion of variance in PA – approximately 77% – remains unexplained through intentions alone [18], hereby signaling the limited predictive capacity of traditional models which may overestimate the influence of rational decision-making on behavior [20, 21].

**Fig. 1:**
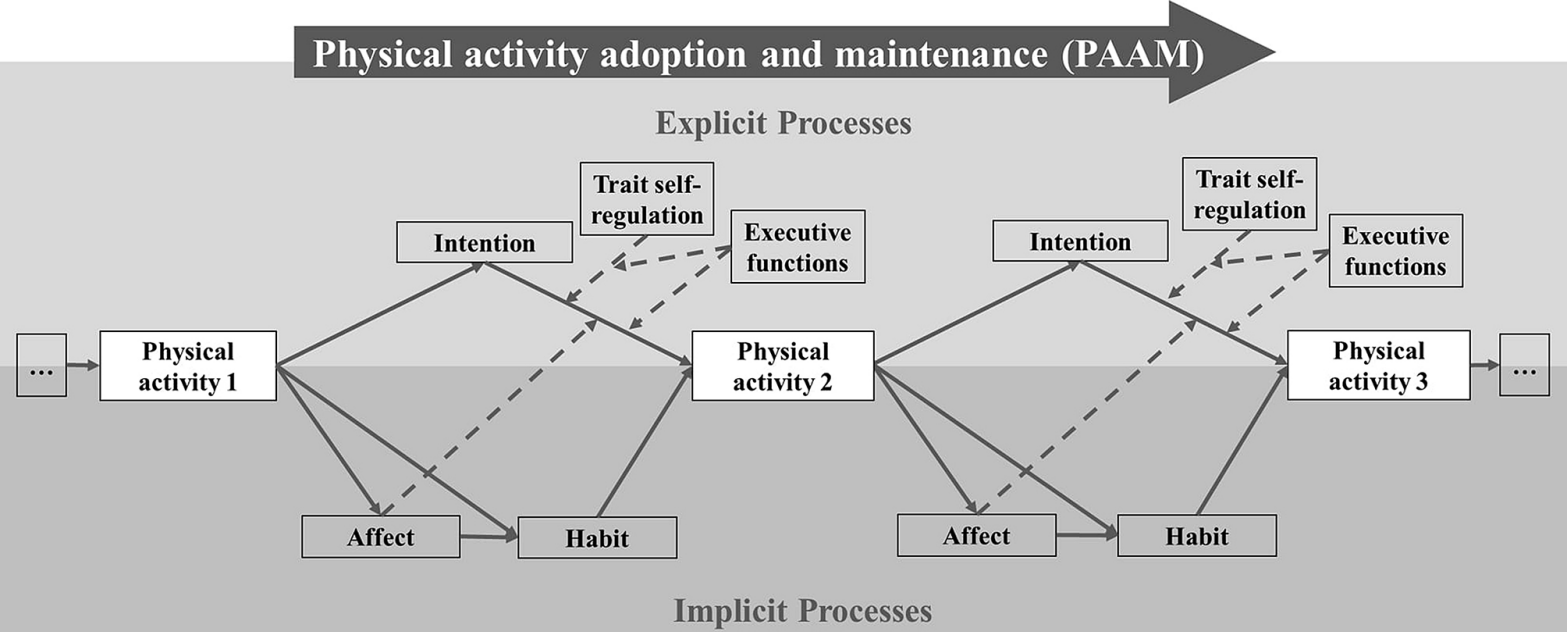
The physical activity adoption and maintenance model (PAAM)

As a consequence, dual-process models have become a useful framework for explaining and forecasting PA behaviors recently [22]. However, from the perspective of dual-process theories, PA is controlled by two processing systems: explicit (also known as reflective) processes and implicit (also known as automatic) processes (we explain these terms in more detail in Sect. 1.1.1 and 1.1.2, respectively). These two systems operate simultaneously and may also interact with one another [23]. Although research on the effects of the interaction between explicit and implicit processes in PA behavior exists, this research is still limited [24, 25]. Therefore, the purpose of the current study is to empirically test the assumptions of the recently developed Physical Activity Adoption and Maintenance model [PAAM model; 26], which combines explicit and implicit processes to explain PA adoption and maintenance (see Fig. 1). In the present paper, we chose this model over other models in the field, because the PAAM model allows for empirical testing of theoretical predictions.

### The physical activity adoption and maintenance model

The PAAM model proposes that human behavior is governed by two distinct information-processing systems that are characterized by their degree of automaticity and reflectivity [27, 28]: implicit and explicit processing systems. Both systems operate simultaneously and interact with each other. Implicit processes serve as the default response upon which explicit processes are based. However, the explicit processes can override the behavioral impulses of the implicit processes if the person has sufficient cognitive resources available [29]. Implicit and explicit processes can be concordant or conflicting, resulting in either a facilitation or an inhibition of PA behavior [30]. In accordance with the Reflective-Impulsive Model [28], the interplay between the implicit and explicit processes can be described as a competition for control over the overt response [23].

#### Explicit processes and PA

*Explicit processes* refer to the deliberative and intentional cognitive processes that guide behavior, such as decision-making, planning, and goal-setting [31]. These processes are primarily studied within social-cognitive theories, including the Theory of Planned Behavior [22]. In addition, self-regulatory skills, such as trait self-regulation, have been linked to explicit processes and health behaviors [32]. In the PAAM model, explicit processes include PA intention, trait self-regulation, and executive functions (EFs). Although intention is considered a crucial factor in predicting PA behavior in many health behavior models [14], empirical studies have shown that intention alone does not fully explain the variance in PA behavior [16, 33, 34]. This lack of covariation between intention and behavior highlights the need to consider additional factors beyond intention when predicting PA behavior.

Research has shown that self-regulatory skills are crucial for the implementation of intended behaviors, especially when difficulties and barriers are present [35]. Trait self-regulation is one aspect of self-regulatory skills that has been found to be of utmost importance in health-related domains [36]. The PAAM model posits that explicit processes such as trait self-regulation play a vital role in bridging the intention-behavior gap in PA domains, as individuals with higher levels of trait self-regulation are more likely to overcome barriers and resist immediate gratifications in favor of long-term benefits [37]. In contrast, individuals with lower levels of trait self-regulation may find it difficult to regulate their behavior and thus struggle to adopt and maintain PA behaviors [38]. Therefore, developing and enhancing trait self-regulation might be a critical aspect of interventions aimed at promoting PA behavior.

EFs are a set of cognitive processes that facilitate goal-directed behavior, including self-regulation [39]; because of this facilitative nature and because EFs require processing capacity, these functions are a component of the explicit processes. The tripartite model of EFs includes distinct but interrelated domains: inhibition, updating, and shifting [40]. Inhibition involves the suppression of automatic or habitual responses that conflict with a current goal, while updating allows for the active representation and manipulation of relevant goal-related information. Shifting allows individuals to flexibly adapt their behavior to changing circumstances, thus avoiding a rigid and ineffective approach to goal attainment [39]. EFs have been associated with successful self-regulation in various domains, including health behaviors such as PA [37]. However, the specific role of the three EF domains in regulating PA has not been extensively studied [41]. Inhibition has been associated with better emotion regulation during exercise and overcoming negative affective responses [42]. Updating facilitates the active representation and flexible adjustment of PA goals and the means to achieve them, while shifting helps individuals adapt to changing environmental demands and overcome barriers to PA [39]. Further research is needed to determine the relative contributions of the three EF domains to successful PA regulation.

Wilkowski and Robinson [43] propose that both motivation and ability are important factors to consider when predicting self-regulatory outcomes. Trait self-regulation can be viewed as reflecting the motivation or willingness to exert self-regulation across various situations and behaviors [44], whereas EFs represent the cognitive ability to exert self-regulation in a given situation in a more process-oriented way [39]. Thus, it is hypothesized that self-regulation and EFs interact in predicting PA behavior and the intention-behavior relation. Preliminary research by Allom, Panetta, Mullan and Hagger [45] and Pfeffer and Strobach [37] supports this hypothesis, suggesting that higher levels of trait self-regulation may compensate for poorer self-regulatory abilities.

The literature suggests that explicit processes play a crucial role in overriding conflicting impulses, such as negative affect toward PA behaviors or sedentary habits [46, 47]. In contrast, implicit processes may dominate when cognitive resources are limited [38]. Recent studies have highlighted the importance of explicit processes for successful self-regulation in the context of PA behavior [48]. However, the interplay between implicit and explicit processes in predicting PA behavior is still an open question that requires further investigation.

#### Implicit processes and PA

*Implicit processes*, in the context of physical activity and behavior change, are defined as the automatic, non-conscious psychological mechanisms that influence behaviors. Compared to explicit processes, these implicit processes are fast, effortless, and triggered by environmental or internal cues, with rigid and inflexible responses to environmental demands [29, 49]. According to the PAAM model, implicit processes include affective responses and habits [26]. Affect refers to nonreflective, simple, and rapid subjective experiences that support the activation of approach or avoidance behavior and are differentiated on the basis of their valence [50]. Affects are simple automatic valuations of whether something is good or bad and should not be confused with full-fledged emotions (e.g., anger, joy). Positive affective states during PA have been shown to influence future PA [51] and to promote the maintenance of PA behaviors [52]. The PAAM model proposes that affective states influence behavior through at least two mechanisms. First, affective states influence habit formation such that when behavior is associated with positive affective states, habit formation is faster, whereas when behavior is associated with rather negative affective states, habit formation is slower [53]. Second, affective states during the behavior moderate the effects of intention on behavior, such that when the behavior is associated with positive affective states, the intention is more easily enacted, whereas, for the same behavior, if the affects are negative, the individual will have more difficulties translating their intentions (provided they have expressed them) into behavior [54].

Habits are automatic responses to environmental cues that develop through repetition in stable contexts and become less effortful over time [55]. Once formed, habits are enacted without conscious awareness and are therefore resistant to change. Consequently, interventions aimed at promoting PA should focus on developing habits rather than solely increasing PA levels [56]. The PAAM model posits that habit formation is characterized by a gradual shift from explicit to implicit control processes through repeated behavioral displays in stable contexts [57, 58]. While behavior change and PA adoption are primarily driven by explicit processes, behavior maintenance is driven more strongly by implicit processes. In the short term, behavior is more strongly regulated by intentions for individuals with weaker habits, while habits lead to more automatic behavior [64–61]. However, there is just rather recent work on how the interaction between intentions and habits shapes behavior [62] within the domain of PA [63].

### The current study

**Fig. 2:**
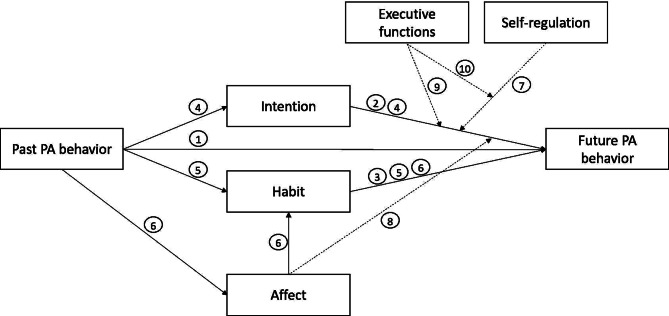
Hypotheses 1 to 10 of the present study in the context of the PAAM model. Note that hypotheses 1 to 8 could be empirically tested in this study

The PAAM model suggests that explicit and implicit processes play a critical role in the adoption and maintenance of PA. While empirical evidence exists for the individual pathways of the model, a comprehensive and combined examination of these pathways and the interplay between the postulated factors has not been conducted in a representative sample. Therefore, this study aims to test the theoretical assumptions of the PAAM model using a multinational sample. The study’s hypotheses (Fig. 2) are derived from the PAAM model, which predicts the positive main effects of [1] past PA behavior [2], intention, and [3] habit on future PA behavior. The study further proposes that [4] intention and [5] habit (at least partially) mediate the association between past and future PA behavior, while [6] affect and habit act as a mediating chain between past and future PA. In addition [7], trait self-regulation [8], affect, and [9] EFs are proposed to moderate the intention-behavior relationship, while [10] EFs and trait self-regulation are proposed to moderate the intention-behavior relationship (i.e., a three-way interaction; higher trait self-regulation may compensate for lower EFs with respect to the intention-behavior relationship).

## Methods

### Participants and procedure

The present study was preregistered on the Open Science Framework (https://osf.io/kv2hn/?view_only=719bd12736fe429c83381275b25a8a16) prior to the commencement of data collection. The primary objective of this study was to recruit adult individuals (≥ 18 years) who did not possess any physical health conditions that could potentially impede their participation in PA. Participation in the study was voluntary, and data were obtained through the administration of online questionnaires and EF tests using Tivian software and the EFS survey, which was distributed at universities and via social media. The questionnaires and tests were provided in English, German, and Italian, and data were collected at two separate time points with a 4-week interval between them. This observational study was completely online. During the initial time of measurement (t1), participants were first provided with informed consent before being instructed to generate a pseudonym (participant code). Subsequently, they were required to provide demographic information and respond to assessments of the PAAM variables, including trait self-regulation, PA behavior at t1, PA intention, PA habit strength, and affective reactions experienced during PA as independent or control variables. The order of these assessments was counterbalanced and randomized. Additionally, participants were directed to four EF tests through provided links. To ensure anonymity, participants were asked to provide a valid email address solely for the purpose of receiving the participation link for the second time of measurement (t2). This email address was stored separately from other data to maintain participant anonymity. After a 4-week interval in which participants had no contact with the present study, they received the survey link for t2 and were instructed to utilize the same participant code generated during t1. During t2, participants were exclusively required to report their PA behavior during the last 7 days as dependent variable..

In the planning phase of this research, a comprehensive power analysis was conducted to ensure the study was adequately powered to detect the effects of interest. Previous meta-analytical evidence suggests that intention and habit exhibit moderate to large effect sizes on behavior change outcomes [18, 57]. However, given the pioneering nature of our study on the assessment of moderation effects in this context – where empirical precedence is limited – we adopted a conservative estimation, assuming a small effect size (f^2^ = 0.05). Using an alpha level of 0.05 and aiming for a statistical power of 80% to detect significant effects among the seven predictors included in the hierarchical linear regression, the calculated required sample size was 295 participants. At t1, the German version of the questionnaire was completed by 482 participants, the English version by 258, and the Italian version by 79. Of these, 251, 122, and 49 participants also answered the German, English, and Italian versions of the questionnaire at t2 (i.e., stayers), respectively, resulting in dropout rates of 47.9% (German), 52.7% (English), and 38% (Italian). Analyses of differences between stayers (i.e., participants that took part in t1 and t2) and dropouts (i.e., participants that took part in t1 exclusively) showed that there were no differences in age (*t* = 1.8; *df* = 817; *p* = 0.8), gender (χ^2^ = 1.1; *df* = 1; *p* = 0.22); PA (*t* = 1. 9; *df* = 817; *p* = 0.06); intention (*t* = 1.5; *df* = 817; *p* = 13), habit (*t* = 1.2; *df* = 817; *p* = 0.23); affect (*t* = 1.7; *df* = 817; *p* = 0.09), and self-control (*t* = -0.7; *df* = 817; *p* = 0.49). The only variable on which dropouts and stayers significantly differed was educational attainment (*t* = 4.7; *df* = 817; *p* < 0.01), indicating that dropouts (*M* = 14.3; *SD* = 4.8) had approximately 1.7 more years of education than stayers (*M* = 12.6; *SD* = 5.7). A total of 422 participants (*M*_*age*_= 25.3, *SD*_*age*_= 10.1; min_age_ = 18; max_age_ = 69; 74.2% female) completed both t1 and t2, and all participants confirmed proficiency in the respective language of the questionnaire. The study was approved by the Ethics Committee of the Medical School Hamburg (reference number MSH-2020/106) and conducted in accordance with the Declaration of Helsinki.

### Measures

#### Demographic information form

Age, sex, nationality, and fluency in the respective questionnaire language were assessed as demographic information.

#### Trait self-regulation

The Brief Self-Control Scale (BSCS; Tangney et al., 2004; German version by Bertrams & Dickhäuser, 2009; Italian version by Gürdere et al., 2022) was applied to measure trait self-regulation. It is a one-dimensional 13-item scale (e.g., “I am able to work effectively toward long-term goals.”), with each item being answered on a 5-point scale (1 = *not at all* to 5 = *very much*). Higher scores indicate higher levels of trait self-regulation. Several studies have supported the psychometric properties of the different language versions of the BSCS [69–66].

#### PA behavior

We measured PA behavior at t1 and t2 with the four items derived from the short form of the International Physical Activity Questionnaire [IPAQ; 67]. Participants were asked to indicate how many times they performed moderate and vigorous PA during the last 7 days, and for how long (in minutes) on average per occasion. The frequency values were multiplied with the average duration per occasion for moderate and vigorous PA [cf., 68]. The final moderate-to-vigorous PA score was calculated by summing up the moderate and vigorous PA minutes per week. The measurement properties of the IPAQ instruments demonstrate satisfactory qualities, comparable to or even superior to those of other established self-report measures [67].

#### PA intention

We assessed PA intention using three items (Ajzen, 1991; Pfeffer, Englert, & Müller-Alcazar, 2020), e.g., “I intend to engage in PA for at least 30 min per day with moderate-to-vigorous intensity”. Each item was rated on a Likert scale ranging from 1 (*strongly disagree*) to 6 (*strongly agree*). The PA intention score is calculated as the sum of the three item responses. The scale shows high internal consistency (e.g., Cronbach’s Alpha = 0.89; Pfeffer, Englert, & Müller-Alcazar, 2020).

#### PA habit strength

To measure the strength of PA habits, we used the four-item subscale of the Self-Report Behavioral Automaticity Index of the Self-Report Habit Index [SRHI; 69, 70]. Items assess how automatically an individual engages in PA (e.g. “Physical activity is something I do without having to consciously remember.”). Each item is rated on a 1 (*disagree*) to 5 (*agree*) Likert type scale. The behavioral automaticity score is obtained by adding up the four item responses. The reliability of the four-item automaticity subscale has been demonstrated in previous research (Gardner et al., 2012).

#### Affective reaction during PA (valence)

One item was administered regarding the pleasantness of the feeling while performing PA [the Feeling Scale; 71]. Participants were asked to rate their affect during moderate-to-vigorous physical activities (“Please assess how you generally feel while exercising with moderate-to-vigorous intensity.”) from very bad (-5) to very good (+ 5). The Feeling Scale has been validated for several languages [cf., 72].

#### Executive functions

We measured EF via the online platform Tatool [73]. Two tests measured the inhibition function, and another two tests measured the updating function. Due to time constraints and the online nature of our study, we did not test EF shifting as a third aspect of executive functioning. The inhibition tests were the Eriksen Flanker task [74, 75] and the Simon task [76, 77], while the updating tests were variants of the *n*-back task [78]. The four tests were inserted into exploratory factor analyses in order to create factor scores for each dimension (i.e., inhibition and updating). This should be used for further analyses in order to solve the task impurity problem; this problem arises because any target EF must be embedded within a specific test context, any score derived from an EF test necessarily includes systematic variance attributable to non-EF processes associated with that specific test context [79]. However, the factor analyses did not provide convincing results with regard to factor loadings (e.g., one inhibition test showed a positive and one a negative loading on the inhibition factor), which prevented us from using these scores for further analyses. For this reason, hypotheses 9 and 10 could not be tested.

### Statistical analyses

The software Statistical Package for the Social Sciences (SPSS) version 27 was used for data screening and analyses. Relationships among study variables were examined with Pearson correlation coefficients. To test the hypotheses, a hierarchical multiple regression analysis was conducted. The criterion wasmoderate-to-vigorous PA at t2. To determine the unique predictive value of each predictor, predictors were entered into the hierarchical regression analysis sequentially as a function of their proximity to the behavior. The decision to employ hierarchical multiple regression analysis in our study was driven by its ability to elucidate the incremental value each predictor adds to the explanation of variance in moderate-to-vigorous PA at t2. This analytical approach allows us to systematically assess the unique contribution of each variable—ranging from past PA to trait self-regulation—while controlling for the influence of variables entered at preceding steps. The sequence of entry for predictors into the hierarchical regression was strategically planned based on their theoretical proximity to the behavior in question. This proximity reflects the degree to which variables are directly involved in the initiation and maintenance of physical activity behaviors. In step 1, past PA was inserted as a predictor in the regression, followed by intention (step 2), habit (step 3), affect (valence; step 4), and trait self-regulation (step 5). The two-way interaction terms intention x trait self-regulation as well as intention x affect were included in step 6 and 7, respectively. Because of the different scaling, continuous variables were centered, and dichotomous variables were dummy coded prior to calculating the two-way interaction terms. PROCESS macro for SPSS [80] was utilized for moderation and mediation analyses (based on 10.000 bias corrected bootstraps). In our hierarchical multiple regression analysis, we employed R-square (R²) and the change in R-square (ΔR²) as key measures to quantify the effect size, thereby assessing the magnitude of the relationships observed. R² provides a measure of the proportion of variance in moderate-to-vigorous PA at t2 that is explained by the predictors included in the model. ΔR², on the other hand, offers insight into the additional variance explained with the introduction of each successive block of predictors, highlighting the incremental predictive value added by factors such as intention, habit, affect, and trait self-regulation. This methodological approach ensures a nuanced understanding of the relative contribution of each predictor, enabling us to systematically evaluate the extent to which each variable – sequentially introduced based on theoretical considerations of their proximity to PA behavior – enhances our model’s explanatory power. Because the proportion of missing data due to non-response (*n*_*t1*_ = 819; *n*_*t2*_ = 422) was relatively high at 48.5%, tests for randomness were performed. Little’s MCAR test was not significant (χ^2^ = 9.2; *df* = 7; *p* = 0.24), indicating a rather random pattern of attrition. In light of this finding, we opted for listwise deletion as our primary strategy for handling missing data. This decision was predicated on the random nature of the missing data and the fact that listwise deletion provides consistent and unbiased estimates under the MCAR condition [81]. Additionally, we considered the application of multiple imputation and other advanced methods for dealing with missing data [82]. However, given that our study achieved sufficient statistical power with the existing sample size, as outlined in Sect. 2.1 regarding power analysis, we concluded that the added complexity and potential biases of imputation methods did not outweigh the benefits in this context.

## Results

### Descriptive statistics

The descriptive results are shown in Table 1. All correlation coefficients were statistically significant except for the correlation between intention and self-regulation. The strongest correlations (the greatest *r* value of our study) were the stability correlation of PA and the correlation between intention and automaticity (both *r* = 0.45). The strongest correlations of predictors with PA were automaticity (t1: *r* = 0.41, t2: *r* = 0.42) and intention (t1: *r* = 0.38, t2: *r* = 0.39).

**Table 1:** Descriptive statistics

	N	M	SD	PA_t2	Intention	Habit	Self-regulation	Affect
PA_t1	422	244.97	256.68	0.45**	0.38**	0.41**	0.12*	0.25**
PA_t2	422	204.00	196.96		0.39**	0.42**	0.20**	0.32**
Intention	422	10.92	5.03			0.45**	0.08	0.33**
Habit	422	10.27	4.46				0.22**	0.43**
Self-regulation	422	40.56	7.15					0.24**
Affect	422	2.53	2.15					

*Note*: N = number of participants; M = mean; SD = standard deviation; PA_t1 = moderate to vigorous physical activity at t1; PA_t2 = moderate to vigorous physical activity at t2

### Regression analysis

The results of the hierarchical regression analysis are shown in Tables 2 and 3. The first step of the regression analysis indicated that the moderate to vigorous PA at t1 significantly predicted about 20.3% of the moderate to vigorous PA at t2. This result supports Hypothesis (1) By adding the variable intention into the regression model, the second step predicted about 25.9% of the behavior at t2; this additional 5.6% of explained variance due to the variable intention was statistically significant. In this sense, the results support the Hypothesis (2) The standardized regression coefficient (β) of past behavior was reduced from 0.45 to 0.35. Additional mediation analyses showed that the indirect effect of past behavior via intention on future behavior was statistically significant (*b* = 0.061; Boot SE = 0.017; *t* = 3.7; df = 1; *p* = 0.02), so Hypothesis 4 is empirically supported.

In the third step, the proportion of explained variance increased significantly to 29.5% by including habit in the regression model. The significant increase in explained variance was 3.7%. This result supports Hypothesis 3. From Step 2 to Step 3, the standardized regression coefficients of past behavior went down slightly. The results of additional mediation analyses indicated that the indirect effect of past behavior via habit on future behavior was significant (*b* = 0.046; Boot SE = 0.017; *t* = 2.7; df = 1; *p* = 0.04), so Hypothesis 5 is supported.

**Table 2:** Summary of the hierarchical regression

Model	R	R^2^	Corr. R^2^	ΔR^2^	ΔF	df1	df2	p
1	0.450	0.203	0.201	0.203	106.8	1	420	< 0.01
2	0.509	0.259	0.255	0.056	31.6	1	419	< 0.01
3	0.544	0.295	0.290	0.037	21.9	1	418	< 0.01
4	0.554	0.306	0.300	0.011	6.6	1	417	0.01
5	0.560	0.313	0.305	0.007	4.1	1	416	0.04
6	0.560	0.313	0.303	0.000	0.1	1	415	0.72
7	0.572	0.327	0.316	0.014	8.6	1	414	< 0.01

*Note*: R = Multiple correlation coefficient; R^2^ = squared multiple correlation coefficient; corr. R^2^ = corrected multiple correlation; ΔF = difference of the F-value; df1 = degrees of freedom in numerator; df2 = degrees of freedom in denominator; p = probability value

**Table 3:** Regression coefficients of the hierarchical regression

Step	IV	b	std. error	Beta	t	p
1	Intercept	119.4	11.9		10.1	< 0.01
	PA_t1	0.3	0.0	0.45	10.3	< 0.01
2	Intercept	28.3	19.8		1.4	0.15
	PA_t1	0.3	0.0	0.35	7.8	< 0.01
	Intention	10.0	1.8	0.26	5.6	< 0.01
3	Intercept	-28.3	22.8		-1.2	0.22
	PA_t1	0.2	0.0	0.29	6.3	< 0.01
	Intention	6.9	1.9	0.18	3.7	< 0.01
	Habit	9.9	2.1	0.22	4.7	< 0.01
4	Intercept	-138.6	48.6		-2.8	< 0.01
	PA_t1	0.2	0.0	0.29	6.2	< 0.01
	Intention	7.1	1.8	0.18	3.9	< 0.01
	Habit	8.9	2.1	0.20	4.1	< 0.01
	Self-regulation	3.0	1.2	0.11	2.6	0.01
5	Intercept	-175.0	51.7		-3.4	< 0.01
	PA_t1	0.2	0.0	0.28	6.1	< 0.01
	Intention	6.5	1.9	0.17	3.5	< 0.01
	Habit	7.7	2.2	0.17	3.4	< 0.01
	Self-regulation	2.5	1.2	0.09	2.2	0.03
	Affect	8.6	4.3	0.09	2.0	0.04
6	Intercept	-141.8	106.7		-1.3	0.18
	PA_t1	0.2	0.0	0.28	6.1	< 0.01
	Intention	3.5	8.7	0.09	0.4	0.69
	Habit	7.7	2.2	0.17	3.4	< 0.01
	Self-regulation	1.7	2.7	0.06	0.6	0.53
	Affect	8.8	4.3	0.10	2.1	0.04
	Inter_SR x Int	0.1	0.2	0.09	0.4	0.72
7	Intercept	-23.5	113.4		-0.2	0.84
	PA_t1	0.2	0.0	0.27	6.0	< 0.01
	Intention	-11.0	10.0	-0.28	-1.1	0.27
	Habit	7.0	2.2	0.16	3.1	< 0.01
	Self-regulation	3.0	2.7	0.11	1.1	0.26
	Affect	-11.1	8.1	-0.12	-1.4	0.17
	Inter_SR x Int	0.0	0.2	-0.04	-0.2	0.87
	Inter_Aff x Int	2.2	0.8	0.61	2.9	< 0.01

*Note*: IV = independent variable; b = unstandardized regression coefficient; std. error = standard error; Beta = standardized regression coefficient; t = t-value; p = probability value; PA_t1 = moderate to vigorous PA at t1; Inter_SR x Int = Interaction between trait self-regulation and intention; Inter_Aff x Int = Interaction between affect and intention

In the fourth step, the inclusion of the variable self-regulation significantly increased the amount of explained variance by 1.1%. From Step 3 to Step 4, the standardized regression coefficients of past PA, intention, and automaticity were only marginally affected.

In the fifth step, the inclusion of affect in the regression significantly increased the amount of explained variance by 0.7%. The effects of the other variables in the regression remained relatively stable. However, additional mediation analyses revealed that the threefold indirect effect of past behavior via affect and habit on future behavior was statistically significant (*b* = 0.013; Boot SE = 0.005; t = 2.6; df = 1; *p* = 0.04), indicating that the effect of past behavior on affect, the effect of affect on habit, and the effect of habit on future PA were all significant. In this sense, Hypothesis 6 was empirically supported.

In the sixth step, the interaction between self-regulation and intention did not significantly contribute to the explanation of the variance of future PA. However, by including this interaction in the regression model, the main effects of intention and self-regulation were no longer statistically significant. Hypothesis 7 could not be supported by the results.

**Fig. 3:**
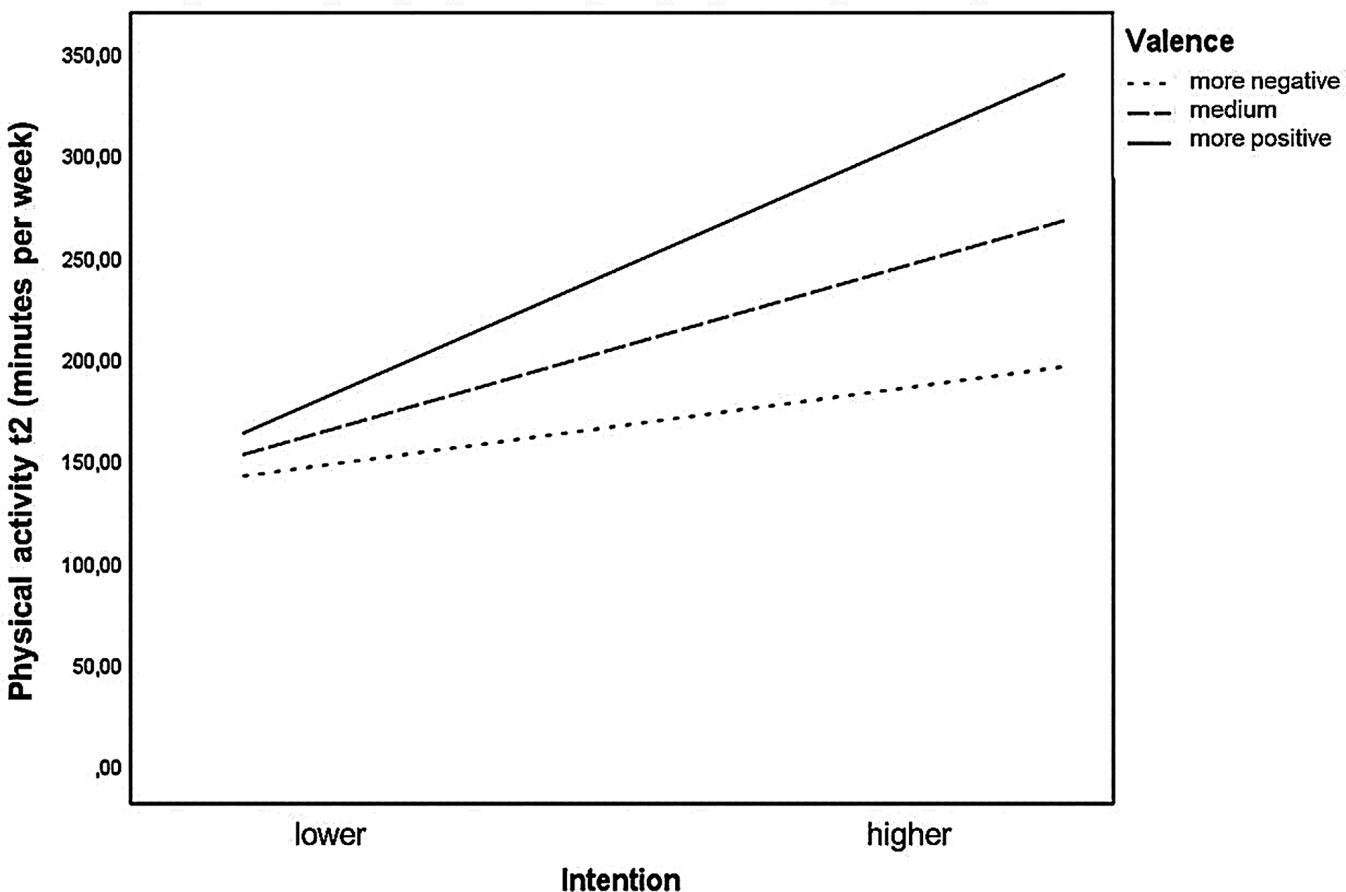
Moderation of the valence of affect on the intention-behavior relationship

In the seventh and final step, including the interaction between affect and intention in the regression significantly increased the amount of explained variance by 1.4%. By including this variable in the regression, the main effect of affect was no longer statistically significant. Figure 3 illustrates the interaction effect. When the valence of affect is rather negative, the effect of intention on behavior is low, and the slope is not statistically significant (*b* = 4.1; SE = 2.2; *t* = 1.9; df = 1; *p* = 0.063). However, when the valence of affect was rather positive, the behavioral intention had a significant effect (*b* = 13.6; SE = 2.5; *t* = 5.5; df = 1; *p* < 0.01). Thus, Hypothesis 8 can be accepted. In this last step of the regression analysis, only past behavior, habit, and the interaction between affect and intention had a statistically significant effect on future PA.

## Discussion

The main purpose of this study was to provide the first comprehensive empirical test of the PAAM model, based on a multi-national sample. At least, ten hypotheses can be derived from the PAAM model, of which eight were subjected to empirical testing in this study. Results confirmed seven of the eight tested hypotheses. The implications of each confirmed hypothesis are discussed in the subsequent sections.

Consistent with the assumptions of the PAAM model, our findings provide empirical evidence for the predictive role of past PA, intention, and habit on PA behavior (hypotheses 1–3). Past PA has been widely recognized as a strong predictor of future PA [83, 84], although it is not necessarily a stable behavior [85]. Intention, which reflects the motivation to engage in PA, is a crucial factor in predicting PA, as demonstrated by previous research [34]. Our study further supports the notion that intention is a more relevant predictor of PA behavior than past behavior. Moreover, our findings indicate that habit is a significant predictor of PA behavior, even after accounting for the influence of past PA behavior and intention, and despite intention and habit showing a relatively high correlation (*r* = 0.45). This suggests that habits play a significant role in regulating PA behavior and that complex behaviors may be regulated by both explicit and implicit processes. It is worth noting that more complex PA behaviors are less likely to be performed automatically due to the need for planning and preparation [49]. Our results could also be interpreted in the context of different phases of behavior change, with intention being more important in the adoption phase and automaticity becoming more dominant in the maintenance phase. This is consistent with the gradual shift over time from explicit to implicit regulation of PA behavior, as proposed by the PAAM model [26]. Future research could explore this idea in longitudinal studies with longer observation periods, as habit formation appears to be a protracted process [55].

One of the central assumptions of the PAAM model is that the effects of past behavior are mediated by both explicit (e.g., intention) and implicit processes (e.g., habit and affect) [cf., 26]. These mediation hypotheses were all supported by the results of the regression analysis. Both intention and habit have a unique effect on future behavior as well as a mediating effect of past behavior. From the perspective of the PAAM model, the mediation of PA intention (hypothesis 4) means that past behavior serves as a basis for decision-making through the formation of new intentions and the consolidation of existing ones. Studies within the context of the Theory of Planned Behavior support this hypothesis, namely that past behavior is a significant predictor of both intentions and behavior [34]. The second mediation path via habit (hypothesis 5) supports the assumption that a habit is formed through behavior repetition in stable situations. In this sense, habit is both the product of past behavior and a predictor of future behavior. This hypothesis is also supported by a large number of studies [cf., 86, 87]. Hypothesis 6 was also supported, with affect and habit acting as a mediator chain between past and future PA, highlighting the role of positive affective reactions in the habit formation process.

In contrast to the predictions of the PAAM model, the role of self-regulation in the current study was found to be more complex (Hypothesis 7). Specifically, when trait self-regulation was included in the regression analysis along with past behavior, intention, and habit, it significantly increased the amount of explained variance, contradicting the predictions of the PAAM model [26]. However, when the interaction between self-regulation and intention was included in the regression, the effect of intention disappeared and the interaction was not statistically significant, suggesting that the effect of the interaction may be confounded by the main effects of intention and self-regulation. These findings are inconsistent with those of a previous study [38], which found a significant interaction between self-regulation and intention in predicting PA behavior.

Trait self-regulation has been found to be associated with health and PA behavior, but the mechanisms underlying this association are not fully understood [88, 89]. Some authors have suggested that individuals with higher levels of trait self-regulation may be better at automating their behavior by forming new habits [90, 91]. Consistent with this hypothesis, studies have shown that PA habits mediate the effect of self-regulation on both effortful inhibition and behavioral automaticity in the context of PA [89]. Furthermore, Pfeffer and Strobach [92] found that trait self-regulation was positively associated with PA automaticity (i.e., habit) and enhanced PA behavior through an indirect effect via this variable. These results provide empirical evidence for the suggestion that individuals with better self-regulation can enact PA behavior more automatically and effortlessly by relying on stable habits; they do not need to exert much effort and self-regulation (e.g., for deciding whether or not to be physically active or for actively inhibiting strong temptations that restrain them from being physically active). This assumption is supported by the positive correlation (*r* = 0.22) between trait self-regulation and habit in our study. Future studies should examine this additional path within the complex assumptions of the PAAM model.

Focusing on classic models to predict PA behavior (e.g., theory of planned behavior [14]), would not be sufficient to explain the present findings. Since these models mainly focus on explicit processes, they would overestimate the impact of these processes. In turn, they would not represent the importance of implicit processes (e.g., affect), which was demonstrated in the present study, among others. Alternatively, the role of affect in the PAAM model has been confirmed by the findings of this study. The results suggest that affect experienced during past PA behavior influences the development of habits, which in turn influence future PA behavior. Positive affect during PA behavior accelerates the development of habits, while negative affect slows it down. Similar results were found in the study by Weyland, Finne, Krell-Roesch and Jekauc [93], where positive affective states at the end of an exercise session supported habit formation. Moreover, the results of this study provide empirical support for the PAAM model’s hypothesis 8, which suggests that positive affective states facilitate the implementation of intentions for PA. The significant interaction effect between intention and affect observed in this study suggests that individuals with higher levels of both intention and positive affect engage in PA behaviors 150 min more per week than individuals with higher intention but lower levels of positive affect. In this sense, the effect of intention on PA behavior appears to be highly dependent on the level of positive affect. While the study by Kwan and Bryan [94] was able to confirm the interaction effect, the study by Finne, Nigg, Weyland, Sauzet, Wienke and Jekauc [95] did not find empirical support. Nonetheless, a review by Rhodes, Cox and Sayar [19] indicated that affect-based variables, such as affective attitudes, can moderate the intention-behavior relationship. Further research is necessary to elucidate the complex interaction between affect and other variables in the PAAM model [19].

### Strengths and limitations

One of the strengths of the current study is the relatively large and multinational sample, which enhances the generalizability of the findings. Additionally, the study’s strictly hypothesis-driven approach and prospective design are noteworthy strengths. Moreover, all predictors were recorded before the criterion, so the criterion of temporal precedence was given.

However, several limitations of the study should also be acknowledged. First, the inclusion of only two points of measurement may restrict our ability to capture the nuanced within-person variations that are pivotal for a more dynamic analysis of the intention-behavior relationship. In many instances, the stability of intention has been identified as a crucial predictor of behavior, serving as a moderator in the intention-behavior dynamic [96]. A more detailed exploration of these fluctuations would necessitate intensive longitudinal studies, employing methodologies such as ecological momentary assessment to garner a deeper understanding of these processes over time [97]. Second, both times of measurements were only four weeks apart, which allows the investigation of PA adaptation and maintenance across only a limited time range. Third, the attrition rate (38-53%) is also a weakness of this study that could have created bias in the results. However, the results show that the attrition was rather random. Fourth, PA behavior was assessed retrospectively and subjectively, which may have led to social desirability effects. Fifth, the study mainly included a younger sample with high education. Therefore, external validity of the results is limited to this specific population. Sixth, we acknowledge the inherent limitations of the measurement tools employed to assess implicit and explicit processes. While we have utilized the most validated instruments available, these tools may not fully capture the nuanced dynamics of these processes, potentially impacting the depth of our insights into the interplay between intentions, habits, and physical activity behavior. Lastly, the four EF tests used in the study could not be used to test some of the hypotheses due to technical limitations in the data collection process. Future studies should consider using soundproof cabins and conducting assessments in laboratory settings to minimize such limitations.

### Implications for research and practice

Future research should consider employing longitudinal designs with multiple times of measurement to observe PA behavior over extended time periods, such as the process of habit formation and its impact on future PA behavior. The interplay of implicit and explicit processes can also be observed in these studies, which is a central tenet of the PAAM model. One approach is to use ambulatory assessment, which involves the flexible recording of both behavior and its determinants using smartphones in real-time situations [98]. Ambulatory assessment has been shown to be a reliable and valid method for assessing PA behavior and its determinants [99], and it provides more ecologically valid and contextually rich data than traditional laboratory-based assessments. Therefore, future studies could utilize ambulatory assessment to provide a more comprehensive understanding of the complex interplay between implicit and explicit determinants of PA behavior.

The findings of this study have important implications for practice, particularly for interventions aimed at increasing PA behavior. While the findings of this study underscore the PAAM model’s emphasis on the integration of explicit and implicit processes for enhancing PA behavior change, it is important to acknowledge that this dual-process approach resonates with the principles of several other behavior change models. This convergence suggests a broader applicability of interventions that engage both conscious planning and automatic behavior patterns across different theoretical frameworks. Specifically, interventions that incorporate strategies such as goal setting, planning, and self-monitoring may target explicit processes [100], while interventions that incorporate strategies such as habit formation, environmental cues, and automaticity may target implicit processes [101, 102]. Furthermore, the finding that affect was a significant predictor of PA behavior suggests that interventions should aim to change individuals’ affective states. This could be achieved through various strategies such as promoting perceived competence, social interaction between participants, novelty experiences, and physical exertion after the training [103, 104].

## Conclusion

The present study aimed to test a large set of assumptions of the PAAM model in a comprehensive way. The results supported seven of the eight examined hypotheses, indicating that intentions and habits significantly mediated the effects of past behavior on future PA behavior. Additionally, it was demonstrated that the effect of past behavior on future behavior was significant through a mediation chain involving affect and habit. Although the hypothesis that trait self-regulation moderates the intention-behavior relationship was not supported, a significant moderation effect of affect on the same relationship was observed. The current study’s findings have important implications for theory and practice related to PA behavior. The results suggest that interventions targeting both explicit and implicit processes may be more effective in promoting PA behavior change.

## Data Availability

The datasets used and/or analysed during the current study are available from the corresponding author on reasonable request.
